# Genomics-Enabled Analysis of *Puroindoline b2* Genes Identifies New Alleles in Wheat and Related *Triticeae* Species

**DOI:** 10.3390/ijms21041304

**Published:** 2020-02-14

**Authors:** Xiaoyan Li, Yin Li, Xiaofen Yu, Fusheng Sun, Guangxiao Yang, Guangyuan He

**Affiliations:** 1 The Genetic Engineering International Cooperation Base of Chinese Ministry of Science and Technology, Key Laboratory of Molecular Biophysics of Chinese Ministry of Education, College of Life Science and Technology, Huazhong University of Science and Technology, Wuhan 430074, China; yanziahnu@163.com (X.L.); yuixf@hust.edu.cn (X.Y.); fufu4567@126.com (F.S.); 2 Waksman Institute of Microbiology, Rutgers, the State University of New Jersey, 190 Frelinghuysen Road, Piscataway, NJ 08854, USA; yl737@waksman.rutgers.edu

**Keywords:** wheat, wheat genome, kernel hardness, *Puroindoline*, *Puroindoline b-2* variants, genotype-to-phenotype association, synteny, phylogenetic analysis

## Abstract

Kernel hardness is a key trait of wheat seeds, largely controlled by two tightly linked genes *Puroindoline a* and *b* (*Pina* and *Pinb*). Genes homologous to *Pinb*, namely *Pinb2*, have been studied. Whether these genes contribute to kernel hardness and other important seed traits remains inconclusive. Using the high-quality bread wheat reference genome, we show that PINB2 are encoded by three homoeologous loci *Pinb2* not syntenic to the *Hardness* locus, with *Pinb2-7A* locus containing three tandem copies. PINB2 proteins have several features conserved for the *Pin*/*Pinb2* phylogenetic cluster but lack a structural basis of significant impact on kernel hardness. *Pinb2* are seed-specifically expressed with varied expression levels between the homoeologous copies and among wheat varieties. Using the high-quality genome information, we developed new *Pinb2* allele specific markers and demonstrated their usefulness by 1) identifying new *Pinb2* alleles in *Triticeae* species; and 2) performing an association analysis of *Pinb2* with kernel hardness. The association result suggests that *Pinb2* genes may have no substantial contribution to kernel hardness. Our results provide new insights into *Pinb2* evolution and expression and the new allele-specific markers are useful to further explore *Pinb2’*s contribution to seed traits in wheat.

## 1. Introduction

Wheat (*Triticum aestivum* L.) is one of the major stable crops on Earth, feeding around 40% of the world population. Kernel hardness directly affects a set of physical and chemical properties of wheat seeds, such as water absorption, starch damage and flour particles [1,2]. Kernel hardness is one of the key traits in wheat seeds, largely determining milling quality and influencing the end-use qualities [3,4]. The major genetic determinant of wheat kernel hardness is the *Hardness* locus (*Ha*) on chromosome 5DS, harboring three closely linked genes: *Grain Softness Protein-1* (*GSP-1*), *Puroindoline a* (*Pina*) and *Puroindoline b* (*Pinb*). The starch granule-associated protein, Friabilin, determines kernel hardness and is composed of PINA and PINB, encoded by the *Pina* and *Pinb* genes, respectively [5,6,7]. Genetic studies and transgenic studies show that wildtype PINA and PINB proteins confers a soft kernel phenotype [8,9,10,11,12,13,14,15,16]. Certain *Pina* or *Pinb* mutations and their combinations lead to hard kernel phenotypes, with slight variation in kernel hardness between the alleles of *Pina* and/or *Pinb* [17]. As the major causal genes of kernel hardness, the allelic diversity of *Pina*/*Pinb* has been extensively investigated in a wide range of wheat germplasm, revealing 26 alleles of *Pina* and 33 of *Pinb*, as well as a few double null alleles [18,19,20,21,22,23,24,25]. Genotype–phenotype association studies show that the diverse *Pin* alleles are related to the phenotypic variations in kernel hardness.

Due to the functional importance of PINs in wheat, efforts have been made to search for genes that are potentially homologous to *Puroindolines* using the wheat Expressed Sequence Tag (EST) database, resulting in the identification of several transcripts known as *Pinb*-like genes (also known as *Pinb-2v*) [26,27]. Due to their high sequence similarity to *Pinb* (~60%), the molecular characterization of these *Pinb*-like genes and genotype–phenotype correlations have drawn research attentions. Physical mapping of the *Pinb-2v* genes, using several wheat genetic stocks, proves that *Pinb-2v1* is located on chromosome 7D, and *Pinb-2v2* and *Pinb-2v3* are allelic and located on 7B, with *Pinb-2v4* or *Pinb-2v5* located on 7A [27,28,29,30], indicating that these *Pinb-2v* genes might be homoeologous.

Mining for *Pinb-2v* genotypic diversity revealed 23 variants, suggesting less sequence diversity compared to *Pina* or *Pinb* [29,31]. With the development of *Pinb-2v* genotyping primers specific for *Pinb-2v1*, *2v2*, *2v3*, and *2v4* genotype–phenotype association has been studied in several collections of wheat varieties, from which different results were reported [28,29,32,33]. Chen et al. found that *Pinb-2v3* was associated with preferable grain traits and higher kernel hardness compared to those possessing *Pinb-2v2* in soft wheat varieties [26]. The results of *Pinb-2v*’s impacts on kernel hardness have been supported by association analysis using other wheat populations [34]. Another association mapping study that considered population structure and kinship showed that *Pinb-2v* variants were associated with semolina extraction but not kernel hardness [32]. By contrast, a genotype–phenotype study surveying representative U.S. wheat accessions did not support an ascertained role of *Pinb-2v* in kernel hardness [33]. An explanation for this could be that the expression levels of *Pinb-2v* variants (*2v1*, *2v2*, *2v3* and *2v4*) were much lower compared to that of *Pinb*, while varied expression levels were also observed among the *Pinb-2v* variants [35].

It is still inconclusive whether the *Pinb-2v* loci or particular alleles could influence kernel hardness or other kernel traits, even at a lesser extent than *Pina*/*Pinb*. Nevertheless, the prevalent *Pinb-2v* variants identified thus far have limited sequence polymorphisms within open reading frames (ORFs). The *Pinb-2v* genotyping primers were specific for variants *2v1*, *2v2*, *2v3* and *2v4*, with *Pinb-2v2* and *Pinb-2v3* being the most frequently genotyped. Previously, the genomic resources were unavailable to address these critical issues of *Pinb-2v* variants. More recently, owing to the advance in new genomics technologies, such as NRGene deNovoMagic, PacBio single-molecule sequencing, and optical mapping, the contiguous and well-ordered genome assemblies with high-quality annotations are now available in several *Triticeae* species, including bread wheat (*Triticum aestivum*), wild emmer wheat (*Triticum turgidum* spp. *dicoccoides*), the progenitor of the wheat D genome, *Aegilops tauschii,* and that of the wheat A genome *Triticum urartu* [36,37,38,39,40]. These advanced genomics technologies have also substantially contributed to several unambiguously reconstructed, contiguous monocot genomes (maize, broomcorn millet, sugarcane, etc.), as well as to the chromosomal regions with tandem gene clusters and extensive haplotype variations in the gene family [41,42,43,44]. With the bread wheat genome (International Wheat Genome Sequencing Consortium (IWGSC) RefSeq v1.0 for the cultivar Chinese Spring (CS42)), we show that the *Pinb-2v* genes are encoded by five gene models at three homoeologous loci, *Pinb2-7A*, *Pinb2-7B* and *Pinb2-7D*, with the *Pinb2-7A* locus containing three tandemly duplicated copies, *Pinb2-7A1, Pinb2-7A2*, and *Pinb2-7A3*. We propose the new nomenclature of *Pinb-2v* for *Pinb2*, so as to be consistent with the IWGSC RefSeq v1.0 annotation (Table S1) [36]. We establish a series of new PCR-based genotyping primers for *Pinb2* genes. Genome-wide analysis of *Pinb2* genes and applications of the *Pinb2* genotyping method provide new insights into this gene family.

## 2. Results

### 2.1. Genomic and Phylogenetic Analyses of Pinb2 Genes

The DNA and predicted protein sequences of known *Pinb2* variants were used to identify gene models that could be *Pinb*-like genes, annotated in the IWGSC bread wheat genome RefSeq v1.0. Five gene models were identified, two on the B and D genomes (TraesCS7B02G431200 and TraesCS7D02G504800, respectively), with the other three on the A genome in a tandemly manner (TraesCS7A02G514400, TraesCS7A02G514500 and TraesCS7A02G514505). Sequence alignments confirmed their high sequence similarity with the kernel hardness-related genes *Pinb* and *Pina*, as well as the gene encoding *GSP-1* on the *Ha* locus (Figures S1 and S2).

We further expanded our BLAST search to several recently published reference genomes of the *Triticeae* species and identified the *Pinb2* homolog, AET0Gv20021600, in the *Ae. tauschii* genome of accession AL8/78; TuG1812G0700005557.01 in the *T. urartu* genome of accession G1812; and TRIDC7BG068420.1 in the B genome of wild emmer wheat accession Zavitan; as well as a tandem cluster of three *Pinb2* homologous sequences in the A genome of wild emmer wheat, which lack annotations as gene models. The nomenclature of *Pinb2* alleles and the corresponding variants, reported previously, are shown in Table S1 with the suffixes 7A, 7B, and 7D used to indicate the chromosomal locations. The gene models corresponding to *Pinb2* genes from the *Triticeae* species are listed in Table S2.

**Figure 1:**
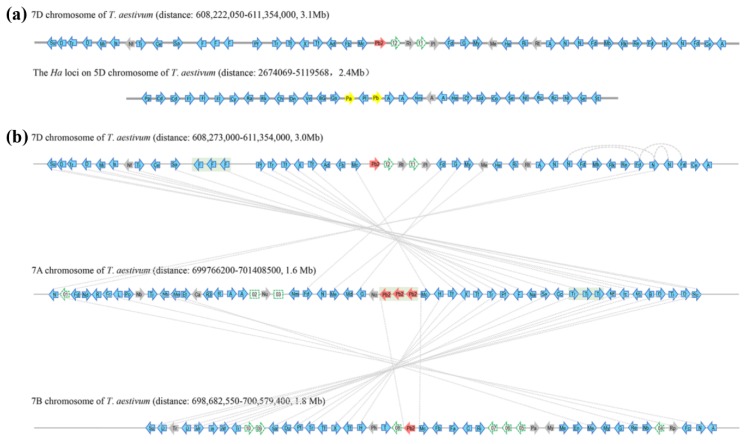
Syntenic alignments of bread wheat chromosomal segments that contains *Pinb2-7D* locus at 7DL and *Ha* locus at 5DS (**a**), and those that contains *Pinb2-7D*, *Pinb2-7A*, *Pinb2-B* at 7DL, 7AL and 7BL, respectively (**b**). Wheat chromosomes are represented as grey horizontal lines, whereas genes along the chromosome are represented as arrowheads. *Pina-D1* and *Pinb-D1* are represented as yellow arrowheads. *Pinb2* are represented as red arrowheads, while the other protein-coding genes with high confidence annotations in the IWGSC RefSeq v1.0 bread wheat genome are represented as blue arrowheads. Homoeologous gene pairs are indicated as dotted lines connecting arrowheads. Tandem duplication of the three *Pinb2-7A* genes is shaded in the grey box. To simplify visualization, only high-confidence protein-coding genes are shown with their orders and orientations along the chromosomal segments, shown as the same in wheat genome assembly. The intergenic regions are not in proportion to the wheat genome assembly. For visualization, only abbreviations of the genes are labeled on the arrowheads, with their full names provided in Table S3.

Due to the high sequence similarity between *Pinb* and *Pinb2*, it has long been considered that *Pinb2* might be associated with kernel hardness. Therefore, we sought to address its relationship with *Pinb* by using synteny alignment. The gene order at the *Ha* locus in the IWGSC RefSeq v1.0 assembly of the bread wheat genome is consistent with previous analyses of the Bacterial Artificial Chromosome (BAC) library containing the *Ha* locus. The *BGGP* gene, encoding b-1-3-galactosyl-O-glycosyl-glycoprotein, and the *GSP-1* gene, encoding Grain Softness Protein-1, are located at the 5′ end of *Ha* (these genes indicated as ‘BG’ and ‘Gs’, respectively, in Figure 1, Table S3) [45,46,47].

Two clusters of *ATPase* genes with a *Nodulin* gene in between are located at the 3′ downstream of *Pina* and *Pinb* (these genes are indicated as ‘A’ and ‘Nm’, respectively, in Figure 1). Comparison of the genes flanking *Pina*/*Pinb* and *Pinb2* failed to identify clear synteny between the *Ha* and the *Pinb2-7D* locus (Figure 1, Table S3). We then analyzed the homologous regions containing the *Pinb2* genes at 7AL, 7BL, and 7DL. Alignment of the *Pinb2* flanking genes revealed a clear collinearity between the segments containing *Pinb2-7A*, *Pinb2-7B*, and *Pinb2-7D* (Figure 1, Table S3). Interestingly, three copies of *Pinb2-7A* with their flanking sequences were arranged in a cluster, suggesting their origin from tandem duplication.

Next, we investigated the collinearity of *Pinb2*-containing segments among the sequenced *Triticeae* species. A comparison of the orthologous segments on chromosome 7A showed collinearity at this region between bread wheat, wild emmer wheat, and *T. urartu*, while only bread and wild emmer wheats have three copies of *Pinb2-7A* (Figure S3a, Table S3). Comparison of the segments on chromosome 7B, between bread and wild emmer wheats, showed a good collinearity between the regions (Figure S3b, Table S3). Furthermore, *TaPinb2-7D1* is contained within a segment that is collinear with an orthologous region of *Ae. tauschii* containing *AtPinb2-7D1* (Figure S3c, Table S3). The *Pinb2-7A* cluster is only detected in polyploidy wheat species, but not in the A-genome and D-genome donor species, *T.urartu* and *Ae. tauschii*, suggesting that the tandem duplication of *Pinb2-7A* could emerge after the polyploidization of wheat.

**Figure 2:**
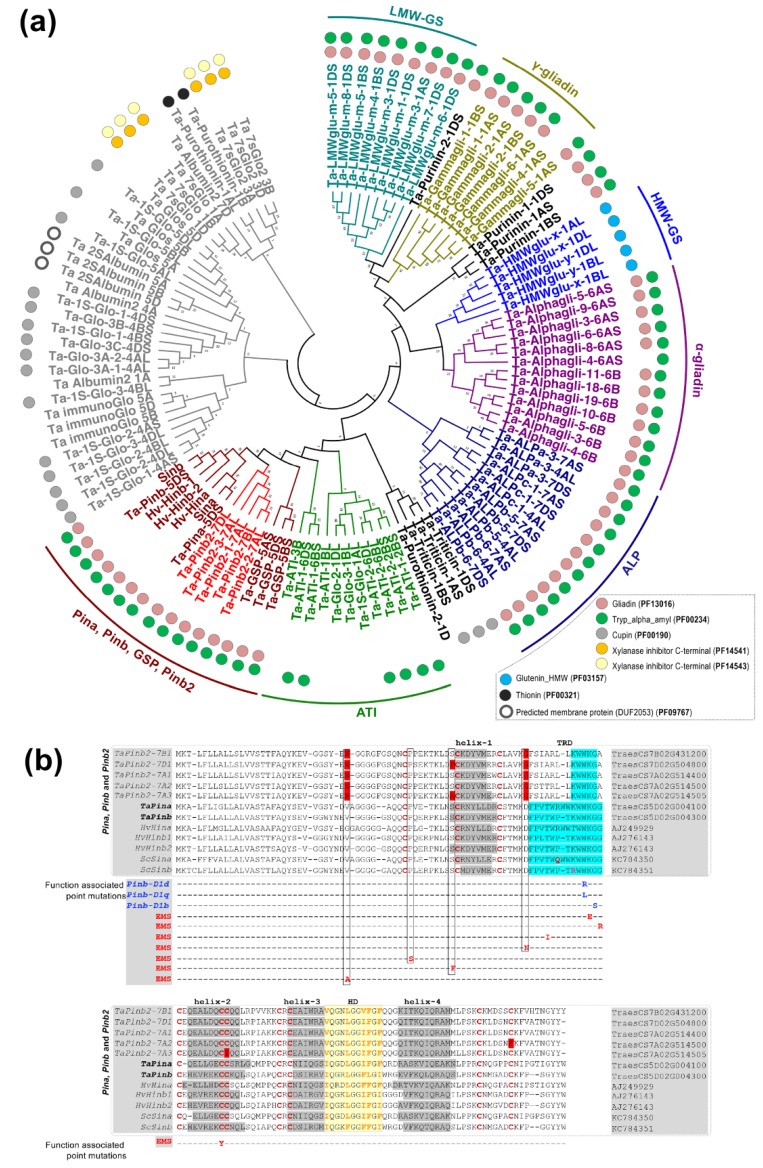
Analyses of phylogeny and protein sequence alignment for PINB2, together with other wheat seed proteins. (**a**) Maximum likelihood (ML) phylogeny of HMW-GS, LMW-GS, α-gliadin, γ-gliadin, purinin, purothionin, avenin-like proteins (ALPs), PINA, PINB and GSP from the bread wheat CS and the homologous proteins of PINA, PINB and GSP from triticale and barley. Results for protein domain analysis using profile hidden Markov Models (HMMER) are shown as color-coded circles. (**b**) Alignment of the amino acid sequences between PINA, PINB, GSP, PINB2, from wheat, barley and triticale, highlights that PINB2 proteins share the cysteine residue backbone, five α-helixes and hydrophobic domain with PINs but lack TRD domain. PINB2 proteins also contain some changes in the conserved amino acids that are important for PINs’ function in kernel hardness (those amino acids shaded in red for PINB2 proteins). α-helixes, hydrophobic domain and TRD are shaded in grey, yellow and blue, respectively. The cysteine residues are shown in red.

While there is a lack of synteny between the genomic segments harboring *Pinb2* and the *Pina*/*Pinb* genes, the *Pinb2* genes indeed have a high sequence similarity with *Pinb*, indicating that *Pinb2* might have the sequence basis for kernel hardness or interactions with other seed proteins. Therefore, we analyzed its phylogeny together with other seed proteins, many of which belong to the prolamin superfamily [48]. The phylogenetic results separated wheat globulins and albumins from those in the prolamin superfamily (Figure 2a, Table S4). PINB2 proteins were clustered together with PINA, PINB and GSP. The puroindoline cluster was grouped together with a cluster largely consisting of α-amylase inhibitors (ATIs). As the repetitive sequences exist in many proteins of the prolamin superfamily and could be problematic for sequence alignment or annotation, we therefore used the domain signatures and conserved cysteine residue patterns to help define the phylogenetic clusters of wheat seed proteins. The results showed that almost all the phylogenetically analyzed prolamin proteins, including PINB2, have Gliadin and Tryp_alpha_amyl domains (PF13016 and PF00234, respectively) except that the α-amylase inhibitors only contain domain PF00234 and high-molecular-weight glutenin subunits (HMW-GSs) contain the HMW domain (PF03157). In addition, the conserved cysteine-rich patterns and number of cysteine residuals can be used as characteristics for defining groups of storage proteins within the prolamin superfamily [43]. For example, HMW-glutenin x- and y-type subunits contain four to six cysteines, while α-gliadin and γ-gliadin have six to eight and eight to eleven cysteines, respectively (Figure S4). Type a-, b- and c- avenin-like proteins (ALPs) have 14, 18–20, and 10–12 cysteine residues, respectively, and some ALPs have effects on dough quality, likely due to the number of cysteine residues for interaction with wheat storage proteins (Figure S4) [49,50,51,52]. Here, PINB2 identified in the bread wheat genome of CS shares the same cysteine-rich backbone consisting of 10 residues as PINA, PINB, and GSP (Figure S4).

We performed a detailed sequence comparison between *Pinb2* and *Pina*, *Pinb*, *puroindoline*-like genes in barley and hexaploid triticale (*Hordoindolines*, *Hin*, and *Secaloindolines*, *Sin*), as well as those *Pinb* alleles that naturally exist or are mutagenized by ethylmethanesulfonate (EMS) with functional changes (Figure 2b) [53,54,55,56,57,58]. The *puroindoline*-like genes in barley include *Hina*, *Hinb1* and *Hinb2* with *Hinb*s, but not *Hina* associated with grain hardness, while the *puroindoline*-like genes in hexaploid triticale (AABBRR), *Sina* and *Sinb*, don’t affect grain hardness [53,56,57,58]. The point mutations identified in these natural and EMS-mutagenized alleles of *Pinb* have been reported to be associated with kernel hardness and were summarized in our previous study [4]. The results showed that PINB2s encoded by all three homoeologous genes had the four helixes and a hydrophobic domain (HD), as with PINA/PINB and their homologs in barley and triticale, but lacked a functional tryptophan-rich domain (TRD), which is key for interactions with polar lipids on starch granule surfaces [59]. Additionally, PINB2s had distinct amino acid residues at several conserved locations that were proved important for PINB’s function in kernel hardness by genetic analyses [4,54,55]. Despite these important sequence differences compared to PINs, PINB2s have the conserved HD domain which could be involved in protein–protein interactions in Puroindolines (Figure 2b) [59,60]. In summary, analyses of PINB2s’s sequence signatures (domains, cysteine residue patterns, etc.) and its phylogeny emphasize the sequence and structural similarities between PINB2 and PINA/PINB but also point out the sequence distinction, suggesting one of the probable reasons for PINB2 not being associated with obvious kernel hardness differences.

### 2.2. Expression Levels of Pinb2

**Figure 3:**
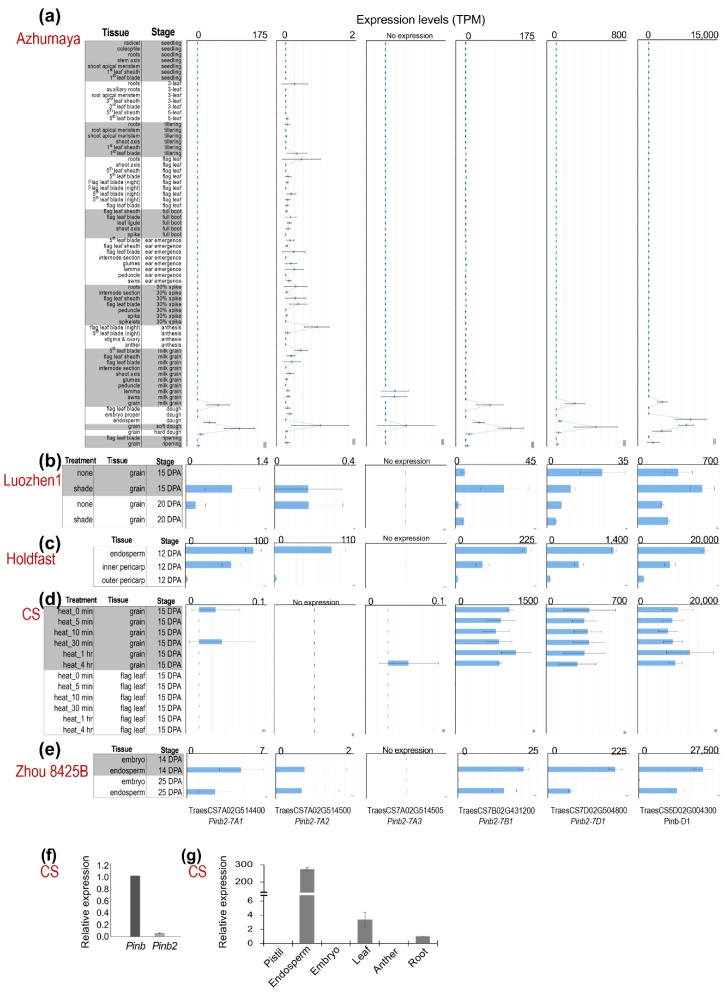
Expression patterns of *Pinb2* genes. Expression levels of *Pinb2* genes and *Pinb-D1* from five publicly available RNA-seq datasets and shown in bar charts (**a**–**e**). The varieties used for RNA-seq analyses are indicated in red, while the tissues, developmental stages and conditions for each RNA-seq experiments are labeled on the left of each panel. Expression levels are shown using TPM and are not normalized across datasets. (**f**) The relative expression levels of *Pinb* and *Pinb2* genes were quantified using qRT-PCR at 28 Days Post Anthesis (DPA) in CS seeds. (**g**) Expression levels of *Pinb2* genes in different tissues from CS proved *Pinb2* genes are expressed specific in seeds. Detailed information of the RNA-seq datasets used are given in Table S5.

High expression levels of *Pina* and *Pinb* during seed development are important for their functions in kernel hardness. We analyzed the *Pinb2* expression dynamics in wheat using the many publicly available wheat RNA-seq datasets. The RNA-seq datasets from five different wheat varieties showed that *Pinb2* genes were expressed at much lower levels compared to *Pinb* (Figure 3; RNA-seq datasets, listed in Table S5 and briefly described in Materials and Methods). For example, *Pinb* was expressed at ~20,000 transcripts per million (TPM) in CS, while *Pinb2-7B1* and *Pinb2-7D1* were expressed at less than 1500 TPM (Figure 3d). Here, to visualize the expression levels of *Pinb2* genes across different RNA-seq experiments or libraries, TPM was used as the expression measure, since it has been suggested to be more consistent and accurate in reflecting the quantitative differences of genes across wheat RNA-seq samples with the consideration that *Pinb2* genes are comprised of a single-exon and short [61,62]. The expression levels of *Pinb2-7A1*, *-7A2*, -7A3, *-7B1* and *-7D1* differed from each other, with *Pinb2-7D1* being the one with a relatively higher expression, followed by *Pinb2-7B1*. *Pinb2-7A3* was expressed at a low level, while *Pinb2-7A1* and *Pinb2-7A2* were expressed at extremely low levels around the RNA-seq expression threshold (>=0.5 TPM), except for their expression in cv. Holdfast (Figure 3c). Previous RNA-seq data of the wheat variety Azhurnaya covers a wide range of tissues and developmental stages, representing a comprehensive wheat expression atlas [62]. Azhurnaya RNA-seq data showed that *Pinb2* genes were highly expressed in several samples of developing seeds, particularly during soft and hard dough stages, with *Pinb2-7D1* most highly expressed followed by *Pinb2-7B1* and *Pinb2-7A1* (Figure 3a). The results also exhibited a very low expression of *Pinb2-7A2* in leaf and root tissues. These RNA-seq data highlights that the expression levels of *Pinb2* genes vary between loci (Figure 3). To explore the *Pinb2* expression across wheat varieties, we visualized the *Pinb2* expression levels together with a reference gene, *TaActin* (TraesCS1D02G274400; Figure S5) [63]. The reference gene TaActin showed relative stable expression for the seed samples within each variety except for cv. Holdfast (Figure S5a). To take into account for potential RNA-seq batch effects between varieties, the TPM expression values of Pinb2 genes were normalized to TaActin. The results showed that Pinb2-7D1 was highly expressed in Holdfast, followed by that in CS, then Azhuranaya and Zhou 8425B, while Pinb2-7B1 was highly expressed in CS, followed by that in Holdfast, then Azhurnaya and Zhou 8425B (Figure S5b). Both TPM expression and relative expression data suggest that the expression levels of *Pinb2* genes vary between varieties. Such varied expression levels of *Pinb2* indicates potential regulatory differences between the loci and varieties, consistent with the much lower sequence similarities in the promoter regions than in the ORFs (Figure S1). Quantitative RT-PCR results validated the lower expression levels of *Pinb2* in comparison with *Pinb* (Figure 3f, Table S6). Our qRT-PCR results also confirmed that *Pinb2* genes were specifically expressed in the developing endosperm of CS (Figure 3g) [35].

### 2.3. Genotyping of Pinb2

PCR methods to amplify the sequences of *Pinb*-like variants in wheat have been established, and these contribute to our understanding of *Pinb*-like variants [26,27,28,29]. Particularly, primers of PCR or derived cleaved amplified polymorphic sequence (dCAPS) assays detecting certain *Pinb2-7B1* or *Pinb2-7D1* variants were reported and have been widely used [32,33,34]. The use of *Pinb2* PCR markers has significantly helped to identify new variants, to study genetic diversity and to facilitate association analysis of *Pinb2* with seed traits, including kernel hardness.

The previous *Pinb2* PCR primers target within the ORF regions, where the sequence variation is much lower compared to the flanking regions, where very few SNPs can be used to distinguish certain variants (Figure S1). With the high-quality reference genomes of several *Triticeae* species, we compared the sequences of *Pinb2* homoeologous genes and designed several new primer pairs for amplifying and detecting *Pinb2*. These PCR primers target *Pinb2*-flanking regions to ensure PCR specificity (Table S7).

**Figure 4:**
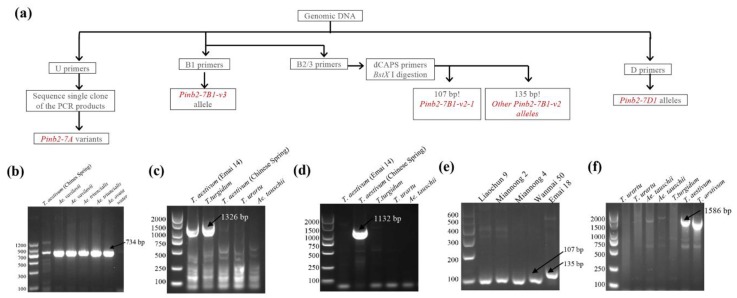
Genotyping of *Pinb2* genes using the new PCR method. (**a**) Diagram describing the workflow of set of PCR primers to genotype *Pinb2-7A*, *Pinb2-7B* and *Pinb2-7D* loci. (**b**) Primer pair U non-specifically amplified *Pinb2* genes encoded by *Pinb2-7A*, *Pinb2-7B* and *Pinb2-7D* loci from several *Triticeae* species. Sizes of DNA markers used are labeled. Specific PCR products are pointed out by black arrowheads. (**c**) PCR primer pair B1 specifically detected the *Pinb2-7B1-v3* allele in bread and durum wheats (*T. aestivum* and *T. turgidum* spp. *durum*). (**d**) Nested PCR primer sets B2/B3 specifically detected the *Pinb2-7B1-v2* allele in bread wheat (*T. aestivum*) Chinese Spring. (**e**) The dCAPS primers-derived PCR amplification and subsequent *BstX* I digestion distinguished the *Pinb2-7B1-v2-1* allele (107 bp) from the other *Pinb2-7B1-v2* alleles (135 bp). (**f**) PCR primer pair D specifically amplified the *Pinb2-7D1* gene in bread wheat.

Among the newly designed *Pinb2* primers, primer pair D can be used to amplify *Pinb2-7D1*, and primer pair U can be used to amplify all three homoeologous *Pinb2* genes (Figure 4f). Primer pair B1 is designed to specifically amplify the *Pinb2-7B1-v3* allele as a ~1326 bp PCR product (Figure 4c). Nested primer pairs B2 and B3 can be used to detect *Pinb2-7B1-v2* alleles [64]. Further, a pair of dCAPS primers (primer pair C) was designed specifically for the *Pinb2-7B1-v2-1* allele. *Bst*X I digestion, after amplification by primer pair C, distinguishes the *Pinb2-7B1-v2-1* allele (107-bp product) from the *Pinb2-7B1-v2* allele (135-bp product), which only has an SNP difference (Figure 4e). Therefore, primer pairs B1, B2/B3 and the dCAPS primers form a sequential pipeline for detecting the alleles of *Pinb2-7B1*.

To validate the primers’ specificity, several accessions of bread wheat (*T. aestivum*), durum wheat (*T. turgidum* spp. *durum*), *Ae. tauschii* and *T. urartu* were used for PCR. Indeed, primer pair B1 amplified the *Pinb2-7B1* genes from bread wheat Emai 14 and durum wheat Ofanto but did not detect the *Pinb2-7B1-v2* allele from CS (Figure 4b,c). The sequencing results of purified PCR products showed that the *Pinb-2v* on chromosome 7B of Emai14 and *T. durum* are *Pinb-B2v3b* and *Pinb-B2v3a*, respectively. The dCAPS primer pair followed by enzyme digestion specifically distinguished the *Pinb2-7B1-v2-1* allele (with a 107-bp product) from other *Pinb2-7B1* alleles (with a 135-bp product, Figure 4e). Additionally, primer pair D only detected the *Pinb2-7D1* genes in bread wheat as designed (Figure 4f), while primer pair U had amplification for all the accessions used (Figure 4b). Overall, these PCR validation results demonstrated the feasibility of our *Pinb2* genotyping pipeline, as illustrated in Figure 4a.

**Figure 5:**
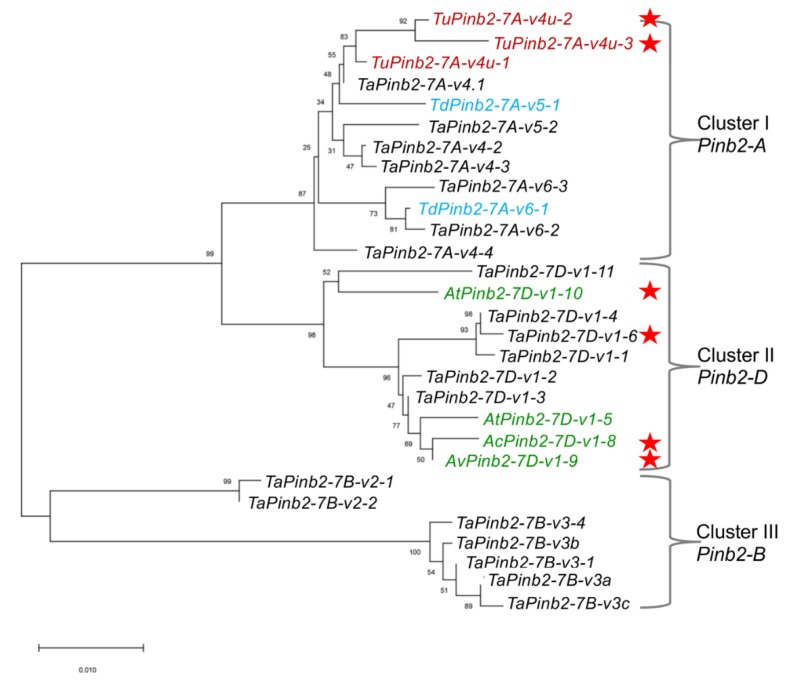
Phylogenetic tree of *Pinb2* variants highlights that these sequences are clustered according to the loci where they are located. Phylogeny of *Pinb2* alleles was constructed using the neighbor-joining method with 1000-time bootstrap and the substitution model of Kimura 2. Different *Pinb2* alleles from different *Triticeae* species in this study are labeled in different colors. The *Pinb2* alleles from durum wheats were labeled in blue color, bread wheat in black, *T. urartu* in red, *Ae. tauschii* in green. The new alleles identified in this study are indicated using red stars.

We then sought to show the usefulness of our *Pinb2* genotyping primers with two examples. First, we explored the diversity of *Pinb2* genes using 70 selected bread wheat accessions and several accessions of *Ae. tauschii*, *Aegilops vavilovii*, *Aegilops triuncialis*, *Aegilops ovata* and *T. urartu*. A new *Pinb2-7D1* allele was amplified from the bread wheat varieties Zhengmai 101, Wanke 06229, Jimai 107, and Laoqimai, designated as *Pinb2-7D1-v1-6*. Four new alleles of *Pinb2-7D*1 were obtained from *Ae. cylindrical*, *Ae. vavilovii*, *Ae. triuncialis* and *Ae. geniculate*, designated as *Pinb2-7D1-v1-8*, *Pinb2-7D1-v1-9*, *Pinb2-7D1-v1-10* and *Pinb2-7D1-v1-11*, respectively (Figure 4b). We sequenced the five new *Pinb2* alleles, *Pinb2-7D1-v1-6*, *Pinb2-7D1-v1-8*, *Pinb2-7D1-v1-9*, *Pinb2-7D1-v1-10*, and *Pinb2-7D1-v1-11*. Sequence alignment and phylogenetic analysis of the new *Pinb2* alleles with the previously identified *Pinb2* sequences showed that the *Pinb2* sequences are clustered according to the A, B and D genomes (Figure 5 and Figure S6).

### 2.4. Association of Pina, Pinb and Pinb2 with Wheat Kernel Hardness

As another application of our *Pinb2* genotyping primers, we genotyped *Pinb2-7B1* and *Pinb2-7D1* loci for 70 Chinese bread wheat varieties, of which the kernel hardness phenotypes and *Pina*/*Pinb* genotypes were reported previously [4]. The 70 varieties were selected based on their highly correlated kernel hardness phenotypes between two field seasons (2016–2017 and 2017–2018) and their uniform *Pina-D1a* genotype, so as to simplify the genotype–phenotype association analysis. The genotypes of *Pinb2-7B1* and *Pinb2-7D1* for these 70 varieties are given in Table S8, with the *Pin* and *Pinb2* frequencies shown in Table 1.

For the *Pinb2-7B1* locus, four alleles (*Pinb2-7B1-v2-1*, *Pinb2-7B1-v3a*, *Pinb2-7B1-v3b* and *Pinb2-7B1-v3c*) were detected and *Pinb2-7B1-v3b* was the most prevalent allele (50%) followed by *Pinb2-7B1-v2-1*. For the *Pinb2-7D1* locus, three alleles (*Pinb2-7D1-v1-3*, *Pinb2-7D1-v1-4*, and *Pinb2-7D1-v1-6*) were identified, with *Pinb2-7D1-v1-4* being the most predominant allele. While all of the 70 varieties uniformly contained the *Pina-D1a* allele, these varieties have three *Pinb* alleles, *Pinb-D1a*, *Pinb-D1b*, and *Pinb-D1p*, of which the latter two are causal for the hard-kernel phenotype due to a point mutation and a lack of PINB protein, respectively (summarized by Li et al.) [4].

Among the 70 varieties, six had a medium-hard kernel (hardness index (HI) between 40 and 60) in one season but were detected as hard kernel (HI > 60) in the other season. These varieties were then classified as mixed kernel-hardness varieties (likely containing multiple *Pin-D1* haplotypes) [65,66,67] and excluded from further analysis. A *Chi*-square independence test showed that *Pinb-D1* was significantly associated with kernel hardness in both seasons (*P* = 3.5 × 10^−11^), and *Pinb2-7B1* was not correlated to kernel hardness (Table 2). *Chi*-square results didn’t support a dependency between *Pinb2-7D1* and kernel hardness although a provisional significance was calculated (*P* = 0.0503). The results here highlight the usefulness of the new *Pinb2* genotyping method and its contribution to association studies of *Pinb2*. Whether *Pinb2-7D1* could play a minor role in kernel hardness, at least in some varieties under particular conditions, requires more large-scale, robust genetic analyses.

## 3. Discussion

*Pinb2* is a group of important genes with several sequence features similar to the kernel hardness-determinant gene *Puroindolines,* though some previous results of association and expression do not support *Pinb2* as playing a major role in controlling wheat kernel hardness. Nevertheless, some genotype–phenotype analyses have indicated that *Pinb2* could be associated with kernel traits [26,34]. Recently, new insights into the functions of *Puroindolines* have emerged, including their abilities to interact with wheat gluten proteins and lipids, possibly through hydrophobic interactions and/or disulfide bonds besides the known TRD–polarlipid interaction [68,69,70]. PINB2 maintains the same cysteine-residue backbone as PIN (Figure 2 and Figure S4). Taken together, these pieces of information justify the need to explore the sequences and function of *Pinb2* in *Triticeae* species, as recently reported for PINs, which was previously mainly reliant on PCR-based genotyping using the limited sequence information of the *Pinb2* ORFs. To facilitate *Pinb2* analysis, we used the high-quality reference genomes of *T. aestivum* and other closely related *Triticeae* species to compare genomic synteny between *Pin* and *Pinb2*, phylogenetically position *Pinb2* among major groups of wheat seed proteins, and develop the new PCR primers for *Pinb2* genes/alleles. Similar types of studies, such as the large-scale identification of immunoresponsive allergens from wheat seed proteins and a genome-wide study of avenin-like proteins or MADS-box genes, were only made possible recently, thanks to the high-quality *Triticeae* genomes [48,51,71].

Using the high-quality wheat genome, we show that *Pinb2* loci are not colinear with the *Hardness* locus on chromosome 5DS, where *Pina* and *Pinb* reside. While there is a lack of synteny between the genomic segments, PINB2 proteins are, indeed, phylogenetically clustered with PINs as well as other ATIs and share the conserved cysteine residue backbone, helixes, and hydrophobic domain with PINs, suggesting an evolutionary relationship between these proteins. Sequence analysis highlights the lack of a functional TRD region and several important amino acids in PINB2, which are required for determining kernel hardness. The contrasting results between the synteny and phylogeny and sequence analyses allow us to speculate the possibility that *Pinb2* or *Puroindolines* might emerge in the progenitor of diploid *Triticeae* species, and a duplicated copy of *Pinb2* might reinsert into the *Ha* locus at chromosome 5DS, or vice versa, followed by an independent evolution for each loci as the divergence of diploid *Triticeae* species and polyploidization to form *Triticum aestivum*. This hypothesis would explain the syntenic alignment results and the emergence of unique TRD in Puroindolines. The evolutionary aspect of *Pinb2* and *Pin* went beyond the scope of our study and requires additional evolutionary and bioinformatics analyses using much broader genomics information, such as those from barley and rye.

Previous studies on the association of *Pinb2-7B* with kernel hardness drew somewhat different results and some results have indicated potential minor effects of certain *Pinb2-7B* alleles on kernel hardness [26,28,32,33,34]. *Pinb2* has also been reported to be associated with other kernel traits in wheat [26,32]. In the present study, we designed the new *Pinb2* PCR markers using the high-quality genome assemblies of bread wheat and several *Triticeae* species and proved the PCR markers to be useful. However, we acknowledged that the *Pinb2* PCR primers might not be perfect. For example, there are a few polymorphisms in the reverse U primer (Figure S1), although it did not affect the annealing of the primers and successful amplification using the wheat materials (Figure 4). Future work will be needed to improve the *Pinb2* genotyping primers toward a higher sensitivity and a broader adaptability for more wheat varieties and *Triticeae* species. With the new *Pinb2* genotyping primers described here, we expanded the genotyping ability of certain *Pinb2-7B* alleles to both three homoeologous *Pinb2* loci. The result of the *Chi*-square test didn’t support the dependency of *Pinb2-7D* or *Pinb2-7B* on kernel hardness, although the association results of *Pinb2-7D* approached significance (*P* = 0.0503). As the major purpose of the present study is to conduct a comprehensive analysis of *Pinb2* genes using the high-quality wheat genome and to proof-of-concept the new *Pinb2* genotyping primers, rather than to draw definite solid conclusions for the association between *Pinb2* and kernel hardness, our interpretation comes with a caveat based on the possibility that the number of varieties may not be sufficient or the varieties used here may not represent broad enough genetic diversity to draw definite solid conclusions for genotype–phenotype association analysis. The marginal significance between *Pinb2-7D* and kernel hardness highlights the necessity of performing further genotype–phenotype association studies for the Pinb2 genes and to expand such appropriate studies to a larger and more representative collection of wheat accessions using the *Pinb2* genotyping primers reported here. The wheat accessions used previously are different between studies, also making it difficult to directly compare the results. Importantly, PINB2 proteins possibly hold the structure and/or sequence basis for interacting with gluten proteins or lipids [68,69,70,72].

More recently, studies provide evidence of PIN–gluten protein aggregation, likely through hydrophobic interactions, particularly the evidence for PIN–gliadin interaction affecting gliadins’ aggregative properties [69,70]. Considering the variations in expression levels between *Pinb2* homoeologous copies observed in the RNA-seq data, it may be possible that certain PINB2 proteins with high expression levels could exert similar properties as PINs, interacting with gluten proteins or lipids through hydrophobic interactions. Thus, our results emphasize future research directions to address the issue whether PINB2 proteins could interact with gluten proteins and/or lipids as PINs, and whether such interactions would exert some effects on kernel hardness or other kernel traits.

## 4. Materials and Methods

### 4.1. Plant Material

A collection of seventy bread wheat varieties from the wheat-producing regions of the Yellow and Huai Valleys and Yangtze Valley of China, provided by the Institute of Crop Breeding and Cultivation of the Hubei Agricultural Academy of Science, and the Institute of Crop Sciences, Chinese Academy of Agricultural Sciences, was used for genotyping *Pinb2* genes and for interrogating their association with kernel hardness using the new genotyping primers developed in the present study (Table S8). The kernel hardness phenotypes of these varieties, collected in the 2016–2017 and 2017–2018 field seasons, have been reported previously [4]. In both seasons, they were planted in the experimental field of Huazhong University of Science and Technology with a randomized complete block design consisting of five 200 cm long rows per accession.

Several *Triticum* and *Aegilops* species were used to validate the PCR specificity and to explore the genetic diversity of *Pinb2* genes; they include *Aegilops tauschii* (donor of the bread wheat A genome, AA), *Ae. vaviolovii*, *Ae. triuncialis*, *Ae. ovata*, *T. urartu* (donor of the bread wheat D genome, DD; accessions G1937 and G1906) and tetraploid *Triticum turgidum* spp. *durum* variety Ofanto (AABB). G1937 and G1906 were obtained from USDA-GRIN.

### 4.2. Phenotyping of Kernel Hardness

The kernel hardness index (HI) was determined with the Single Kernel Characterization System (SKCS) 4100 (Perten Instruments North America Inc., Springfield, IL, USA), following the American Association for Cereal Chemists (AACC) approved method as previously described [73]. Briefly, 200 kernels harvested from each replicate field plot were used for measuring HI. The wheat varieties were planted and measured in the 2016–2017 and 2017–2018 seasons, with two replicated plots for the 2016–2017 season and three replicated plots for the 2017–2018 season. These varieties were classified into soft-, medium-, and hard-kernels based on the HI value: varieties with HI less than 40 were the soft-kernel type, while those with HI greater than 60 were the hard-kernel type, with those with a HI value between 40 to 60 were the medium-kernel type.

### 4.3. Identification and Genotyping of Puroindoline-D1 and Puroindoline b-2 Variants

The genotyping of *Pin a* and *b* was described previously [4]. Six *Pinb2* variants reported previously, *Pinb-2v1*, *Pinb-2v2*, *Pinb-2v3*, *Pinb-2v4*, *Pinb-2v5*, and *Pinb-2v6*, were used to identify the gene models encoding *Pinb2* genes. Then, the coding sequences of *Pinb2* gene models and predicted protein sequences were obtained from the Gramene and *Triticeae* Multi-omics Center Website, the latter of which has been established by Shandong Agricultural University (http://202.194.139.32/blast/viroblast.php). The coding and flanking sequences of *Pinb2* genes were aligned and used to design primers specific to particular loci or variants. Specific primers were designed successfully in the light of variant- or loci-specific regions based on alignment (Figure S1, Table S7). Particularly, the conserved regions of the *Pinb2* variants allowed us to design the universal primer pair U. A pair of dCAPS primers was newly designed to identify allelic variation in *Pinb-B2v2-1* on chromosome 7B. Based on a special, single-nucleotide polymorphism of *Pinb-B2v2-1*, dCAPS Finder v2.0 was used to design dCAPS primer pair C for its use together with restriction enzyme *Bst*X I (Fermentas, Waltham, MA, USA) [74]. Using the new primer pairs described here, a pipeline for detecting known *Pinb2* genes and identifying new *Pinb2* variants at all three homoeologous loci will be possible (details in Results, Figure 4).

Genomic DNA samples from three wheat seedlings of each variety were extracted with the Cetyltrimethylammonium Ammonium Bromide (CTAB) method and used for *Pina*/*Pinb* and *Pinb2* genotyping [73]. PCR amplification was done in a 25 μL reaction volume containing 100 ng of genomic DNA, 0.2 μM primers, and 12.5 µL 2×Es Taq MasterMix (CWBIO, Beijing, China) using the the PCR program as follows: 95 °C for 3 min, 34 cycles of 95 °C for 30 s, 55–62 °C 30 s and 72 °C for 30 s, with a final extension of 5 min in a Bio-Rad-T100 thermal cycler. The PCR products were separated by 1.5% (*w*/*v*) agarose-gel electrophoresis and visualized under UV light after ethidium bromide staining. A sequencing service for the purified PCR products (provided by AuGCT company, Beijing, China) was used to validate the *Pina*/*Pinb* and *Pinb-2v* genotypes and to obtain the sequences of the newly identified alleles of *Pinb2* (Table 3).

### 4.4. Synteny Analysis of the Genomic Segments Containing Pinb2 Genes

The gene order and annotation from several high-quality *Triticeae* species were used for syntenic analysis, including *T. aestivum* IWSGC RefSeq v1.0, the *T. urartu* genome, *Ae. tauschii* genome, and wild emmer wheat (*T. turgidum* spp. *dicoccoides*) genome [36,37,38,39]. High-confidence protein-coding genes flanking *Pina*/*Pinb* genes at 5DS, and those flanking *Pinb2* at 7AL, 7BL and 7DL (within ~2–3 Mb), were retrieved from the genomes. The orthologous gene pairs were determined by using reciprocal BLAST (10e-5, the score is greater than 70%, and the matching base is longer than 100 bp) to compare genes between any two of the genomes. Annotations of the genes and their abbreviated names shown in Table S3.

### 4.5. Quantitative Real-Time Reverse Transcription PCR (qRT-PCR)

Quantitative PCR primers were designed to target the gene-specific coding regions for *Pina*, *Pinb* and *Pinb2*, respectively, according to the sequence alignment by DNAMAN v6.0. *Actin* (TraesCS4B01G050600.1) was used as the internal reference gene for qPCR. Due to the high sequence identity within the *Pinb2* coding region, qRT-PCR primers for quantifying *Pinb2* were only able to detect the overall expression levels of all expressed copies at the three loci (Table S6). The total RNA was extracted from different tissues of the wheat cultivar Chinse Spring [75]. The first-strand cDNA was generated from 1 μg RNA using a FastKing RT kit and gDNase (Tiangen, China) and qRT-PCR was conducted using AceQ qPCR SYBR Green Master Mix (Vazyme, Nanjing, China). The data were evaluated between the three replicates by using the relative quantification method (2^−ΔΔCt^).

### 4.6. Analyses of the Phylogeny and Protein Features for Wheat Seed Proteins

The predicted protein sequences from the bread wheat reference genome were used for phylogenetic analysis together with the predicted protein sequences of *Puroindoline* homologs in triticale and barley (namely, *Sina* and *Sinb* from hexaploid triticale, and *Hina*, *Hinb-1* and *Hinb-2* from barley, *Hordeum vulgare*) (Table S4). Domain analysis using profile hidden Markov Models (HMMER) was performed for the predicted protein sequences of *Pinb2* and other seed proteins to identify several protein domain signatures, for instance the Tryp_alpha_amyl domain (PF00234), HMW-GS domain (PF03157) and gliadin domain (PF13016) [76]. Annotations of wheat seed proteins were reported previously by Juhasz et al. [43] and avenin-like proteins (ALP) were annotated by Zhang et al. [51]. The presence of conserved cysteine residual patterns was used as a feature for the subfamily assignment of the prolamin superfamily [47,48]. The phylogenetic analysis followed a detailed protocol [77]. The protein sequences were aligned using MUltiple Sequence Comparison by Log-Expectation (MUSCLE), guided by an unweighted pair-group method with arithmetic means (UPGMA) tree. The phylogenetic tree of the wheat seed proteins was constructed using the maximum likelihood method provided in the MEGA7 software with a 500-time bootstrap using a Jones–Taylor–Thornton (JTT) model [78].

### 4.7. Statistical Analysis

The association of *Pinb* and *Pinb2* loci with kernel hardness phenotypes was determined by a *Chi*-square test of independence.

## 5. Conclusions

In conclusion, *Pinb2* genes consist of five copies rather than three homoeologous genes due to the additional tandem duplicated copies at *Pinb2-7A*. Based on the synteny and phylogeny analyses, *Pinb2* genes likely preserve the sequence features for interacting with gluten proteins through hydrophobic connections but lack the basis for determining kernel hardness, such as the TRD domain. These results are in line with the association analysis results: *Pinb2* genes do not exert major impacts on kernel hardness as *Pina*/*Pinb*. Leveraging the high-quality reference genome of bread wheat, we develop new *Pinb2* genotyping primers and demonstrate their application in identifying new *Pinb2* alleles and in facilitating association studies. The present study exemplifies an application of the high-quality *Triticeae* genomic resources, and the results implicate the areas for further study to unveil *Pinb2’*s function and its potential use in genetic engineering.

## Figures and Tables

**Table 1:** The distribution and frequency of *Pina*, *Pinb* and *Pinb2* for the 70 Chinese wheat varieties.

Genotypes		*Pinb2-7D1*			*Pinb2-7B1*			
		*2v1-4*	*2v1-3*	*2v1-6*	*2v2-1*	*2v3a*	*2v3b*	*2v3c*
*Pina-D1a/* *Pinb-D1a*	Number	19	2	1	6	5	11	0
	Freq. (%)	86.4	9.1	4.5	27.3	22.7	50	0
*Pina-D1a/* *Pinb-D1b*	Number	24	8	2	7	6	17	4
	Freq. (%)	70.6	23.5	5.9	20.6	17.6	50	11.8
*Pina-D1a/* *Pinb-D1p*	Number	9	4	1	6	0	7	1
	Freq. (%)	64.3	28.6	7.1	42.9	0	50	7.1
Total	Number	52	14	4	19	11	35	5
	Freq. (%)	74.3	20	5.7	27.1	15.7	50	7.1

**Table 2:** *Chi*-square analysis of the association between *Pinb*, *Pinb2-7B1* or *Pinb2-7D1* loci with kernel hardness.

Alleles	2016–17			2017–18			*Chi*-Square*
	Soft	Medium	Hard	Soft	Medium	Hard	
*Pinb-D1a*	20	2	0	20	2	0	*Chi*-square = 54.8 *df* = 4 *P* = 3.5 × 10^−11^
*Pinb-D1b*	1	6	21	1	6	21	
*Pinb-D1p*	0	1	13	0	1	13	
*Pinb2-7B1-2v2-1*	6	3	9	6	3	9	*Chi*-square = 3.4 *df* = 6 *P* = 0.753
*Pinb2-7B1-2v3a*	5	1	5	5	1	5	
*Pinb2-7B1-2v3b*	10	4	16	10	4	16	
*Pinb2-7B1-2v3c*	0	1	4	0	1	4	
*Pinb2-7D1-2v1-3*	2	1	8	2	1	8	*Chi*-square = 9.5 *df* = 4 *P* = 0.0503
*Pinb2-7D1-2v1-4*	19	6	25	19	6	25	
*Pinb2-7D1-2v1-6*	0	2	1	0	2	1	

**Table 3:** The information of new *Pinb2* alleles identified in this study.

New Designation	Previous *Pinb-2v* Designation	Species	Accession	NCBI Accession
*TaPinb2-D1-v1-6*	*Pinb-D2v1-6*	*Triticum aestivum*	Zhengmai 101, Wanke 06229, Jimai 107, Laoqimai	MN839440
*AcPinb2-D1-v1-8*	*Pinb-2v1-8*	*Aegilops cylindrical*	na	MN708354
*AvPinb2-D1-v1-9*	*Pinb-2v1-9*	*Aegilops vavilovii*	na	MN708355
*AtPinb2-D1-v1-10*	*Pinb-2v1-10*	*Aegilops triuncialis*	na	MN708356
*AgPinb2-D1-v1-11*	*Pinb-2v1-11*	*Aegilops geniculate*	na	MN708357
*TuPinb2-A1-v4u-2*	*Pinb-2v4u-2*	*Triticum urartu*	G1937 (PI 428230)	MN893165
*TuPinb2-A1-v4u-3*	*Pinb-2v4u-3*	*Triticum urartu*	G1906 (PI 428228)	MN893166

“na” = not applicable.

## Supplementary Materials

Supplementary materials can be found at https://www.mdpi.com/1422-0067/21/4/1304/s1.

## Abbreviations

ATI	α-amylase inhibitor
ALP	avenin-like protein
BAC	bacterial artificial chromosome
BGGP	β-1-3- galactosyl- O-glycosyl-glycoprotein
CS	Chinese Spring
dCAPS	derived cleaved amplified polymorphic sequence
GSP	grain softness protein
Ha	Hardness
HD	hydropbic domain
HMW-GS	High-Molecular-Weight Glutenin Subunits
IWGSC	International Wheat Genome Sequencing Consortium
LTP	Lipid transfer proteins
ORF	open reading frame
PIN	puroindoline
TPM	Transcripts Per Million
TRD	tryptophan-rich domain
UPGMA	unweighted pair group method using arithmetic average
