# Supplementary material for: Genomics-Enabled Analysis of Puroindoline b2 Genes Identifies New Alleles in Wheat and Related Triticeae Species
Source: Int J Mol Sci. 2020 Feb 14;21(4):1304. doi: 10.3390/ijms21041304 (PMC7072932; doi:10.3390/ijms21041304)
Supplement: Supplementary file 1 [file ijms-21-01304-s001.zip › Pinb2v_R1suppl_20200212.docx]

**Supplementary Files for the manuscript:**

Genomics-Enabled Analysis of *Puroindoline b2* Genes Identifies New Alleles in Wheat and Related *Triticeae* Species

**Xiaoyan Li ^1,#^, Yin Li ^2,#^, Xiaofen Yu^1,#^, Fusheng Sun ^1^, Guangxiao Yang ^1,^*, Guangyuan He ^1,^***

^1^ The Genetic Engineering International Cooperation Base of Chinese Ministry of Science and Technology, Key Laboratory of Molecular Biophysics of Chinese Ministry of Education, College of Life Science and Technology, Huazhong University of Science and Technology, Wuhan 430074, China; [yanziahnu@163.com](mailto:yanziahnu@163.com) (X.L.); [yuixf@hust.edu,cn](mailto:yuixf@hust.edu,cn) (X.Y.); fufu4567@126.com (F.S.)

^2^ Waksman Institute of Microbiology, Rutgers, the State University of New Jersey, 190 Frelinghuysen Road, Piscataway, NJ 08854, USA; [yl737@waksman.rutgers.edu](mailto:yl737@waksman.rutgers.edu) (Y.L.)

***** Correspondence: ygx@hust.edu.cn (G.Y.); hegy@hust.edu.cn (G.H); Tel.: +86-027-87792271 (G.H.)

† These authors contributed equally to this work.

**Supplementary Figures:**

**Figure S1.** Sequence alignment of the *Pinb2* genes from the sequenced *Triticeae* species and primers for *Pinb2* genotyping.

**Figure S2.** Sequence similarities between *Pinb2* genes and *Pina*, *Pinb* or *GSP-1* genes.

**Figure S3**. Syntenic alignments of the chromosomal regions flanking the *Pinb2-A1* **(a)**, *Pinb2-B1* **(b)** and *Pinb2-D1* **(c)** genes.

**Figure S4.** Comparison of cysteine residue patterns between representative proteins in the Prolamin protein families.

**Figure S5.** Expression patterns of *Pinb2* in developing wheat seeds.

**Figure S6.** Sequence alignments of the new identified *Pinb2* alleles.

**Supplementary Tables:**

**Table S1.** The new designation of *Pinb2* genes, alleles or variants.

**Table S2.** The gene models correspond to *Pinb2* genes.

**Table S3.** Abbreviations of the genes shown in **Figure2** and **S3** that presents the syntenic alignment results. [provided as Excel file]

**Table S4.** Annotation of the genes from prolamin superfamily, globulin and albumin families used in this study. [provided as Excel file]

**Table S5.** Information about the five publicly available RNA-seq datasets used for *Pinb2* expression analysis.

**Table S6.** Primers for qRT-PCR of *Pinb2*.

**Table S7**. Primers used for *Pinb2* genotyping.

**Table S8.** *Pina/Pinb* and *Pinb2* genotypes for the 70 Chinese wheat varieties and their kernel hardness phenotypes.

**
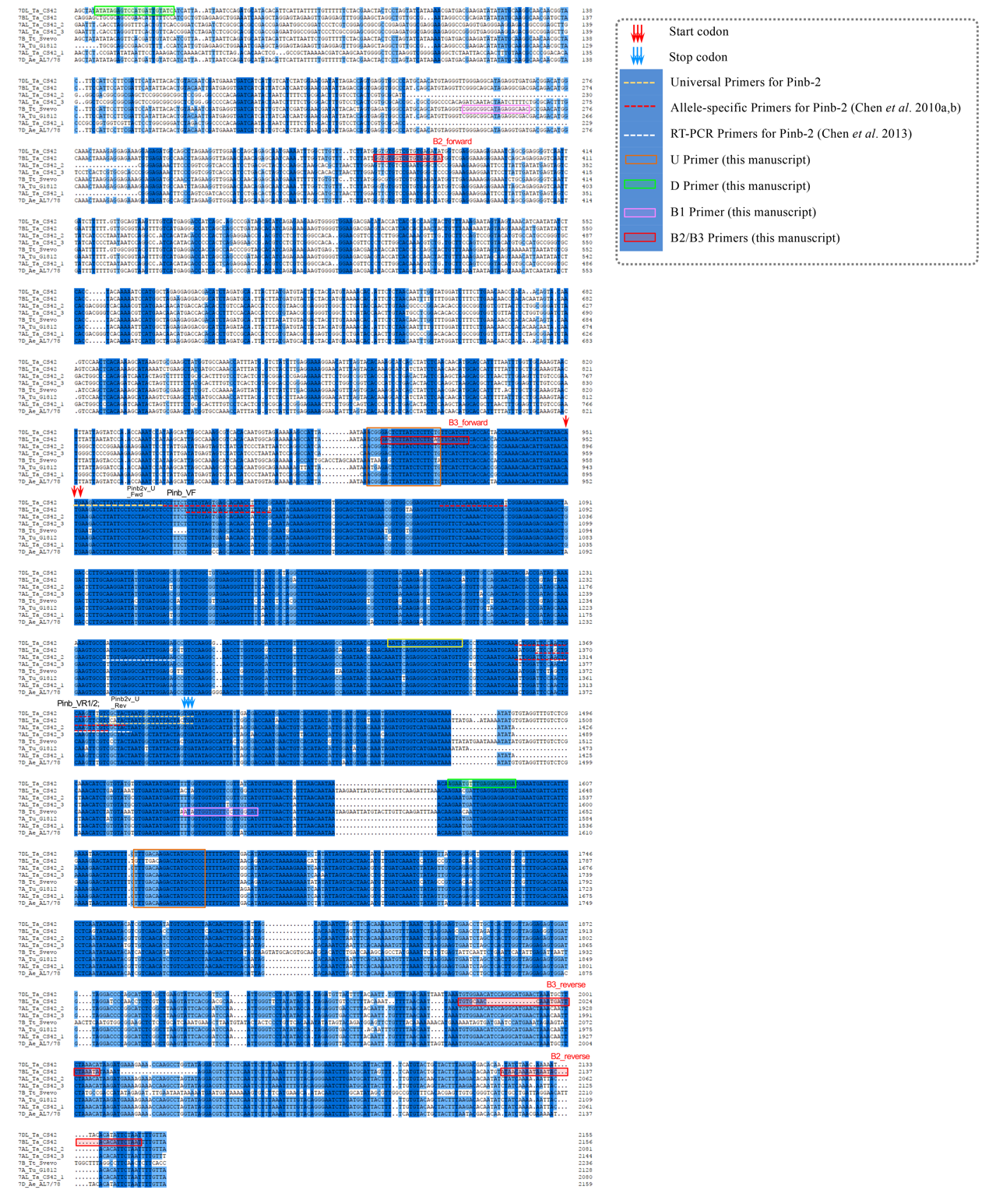
**

**Figure S1.** Sequence alignment of the *Pinb2* genes from the *Triticeae* species, including bread wheat cultivar Chines Spring (*T. aestivum*, abbreviated as CS), durum wheat cultivar Svevo (*T. turgidum*), *T. urartu* accession G1812 and *Aegilops* accession AL7/78 (*Ae. tauschii*). Conserved nucleotides are shaded in dark blue, while nucleotides with some degrees of conservation are shaded in light blue. “7DL_Ta_CS42” and “7BL_Ta_CS42” indicate the genomic regions containing *Pinb2-7D1* and *Pinb2-7B1* genes on 7DL and 7BL, respectively, with “7AL_Ta_CS42_1”, “7AL_Ta_CS42_2” and “7AL_Ta_CS42_3” indicate the genomic regions containing the three copies of *Pinb2-7A* genes, respectively. “7B_Tt_Svevo” indicates the genomic region on 7B containing *Pinb2-7B1* gene in *T. turgidum* (durum wheat ); “7A_Tu_G1812” indicates the genomic region on 7A containing *Pinb2-7A* gene in *T. urartu*; “7D_Ae_AL7/78” indicates the genomic region on 7D containing *Pinb2-7D1* gene in Ae. tauschii. Positions of the primers published previously and used in the present study are labeled as in figure legend (Chen *et al*. 2010, 2011).

**
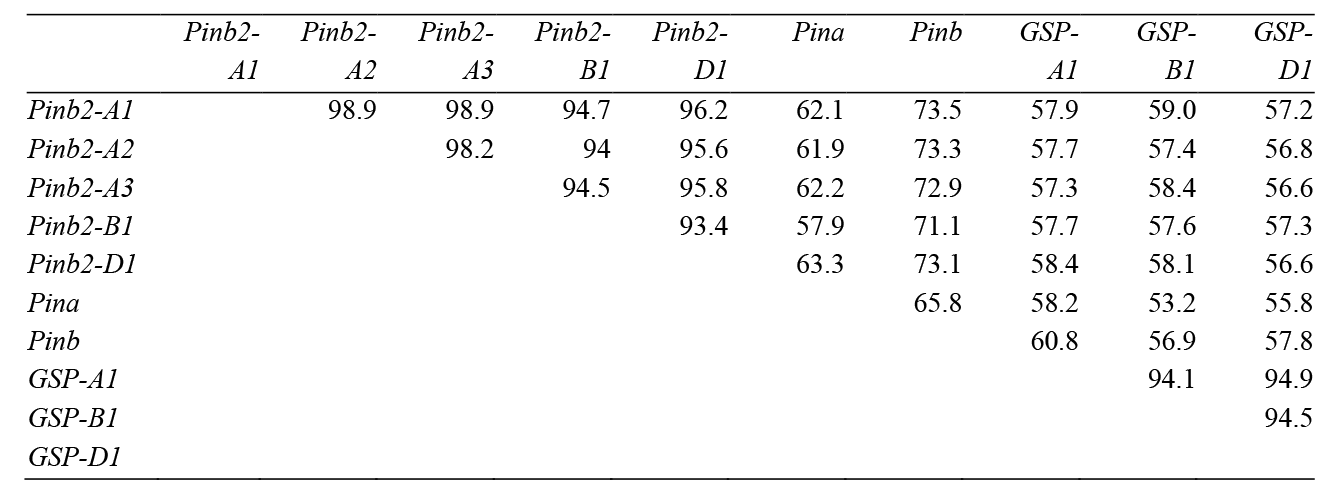
**

**Figure S2.** Sequence homology (percentage of identity) between *Pinb2* genes and *Pina*, *Pinb* and *GSP-1*. Percentage of sequence identity was determined for each pair of gene using ClustalW. Only the Open Reading Frame (ORF) regions were used.

**
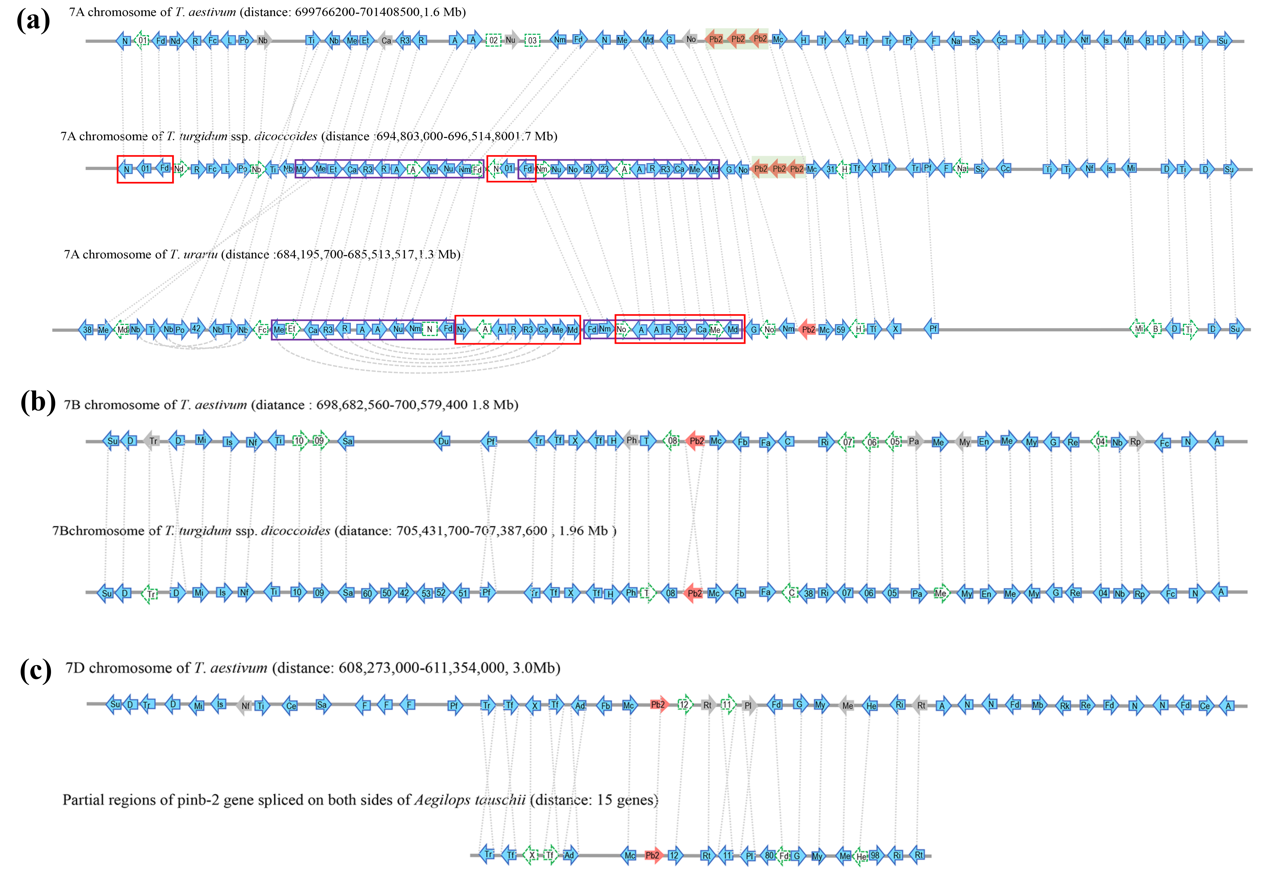
**

**Figure S3**. Syntenic alignments of the chromosomal regions flanking the *Pinb2-A1* **(a)**, *Pinb2-B1* **(b)** and *Pinb2-D1* **(c)** genes. **(a)** The genomic regions containing *Pinb2-A1* genes are compared between bread wheat (*T. aestivum*), wild emmer wheat (*T. turgidum* spp. *dicoccoides*) and the wheat A-genome progenitor *T. urartu*. **(b)** The genomic regions containing *Pinb2-B1* genes are compared between bread wheat and wild emmer wheat. **(c)** The genomic regions containing *Pinb2-D1* genes are compared between bread wheat, wild emmer wheat and the wheat D-genome progenitor *Ae. tauschii*. *Pinb2* are represented as red arrowheads, while the other protein-coding genes with high confidence annotations in the IWGSC v1.0 bread wheat genome are represented as blue arrowheads. Homoeologous gene pairs are indicated as dotted lines connecting arrowheads. Tandem duplication of the three *Pinb2-A1* genes is shaded in grey box. To simplify visualization, only high-confidence protein coding genes are shown with their orders and orientations along the chromosomal segments shown as the same in wheat genome assembly. The intergenic regions are not in proportion to the wheat genome assembly. For visualization, only abbreviations of the genes are labeled on the arrowheads, with their full names provided in Supplementary TableS3.

**
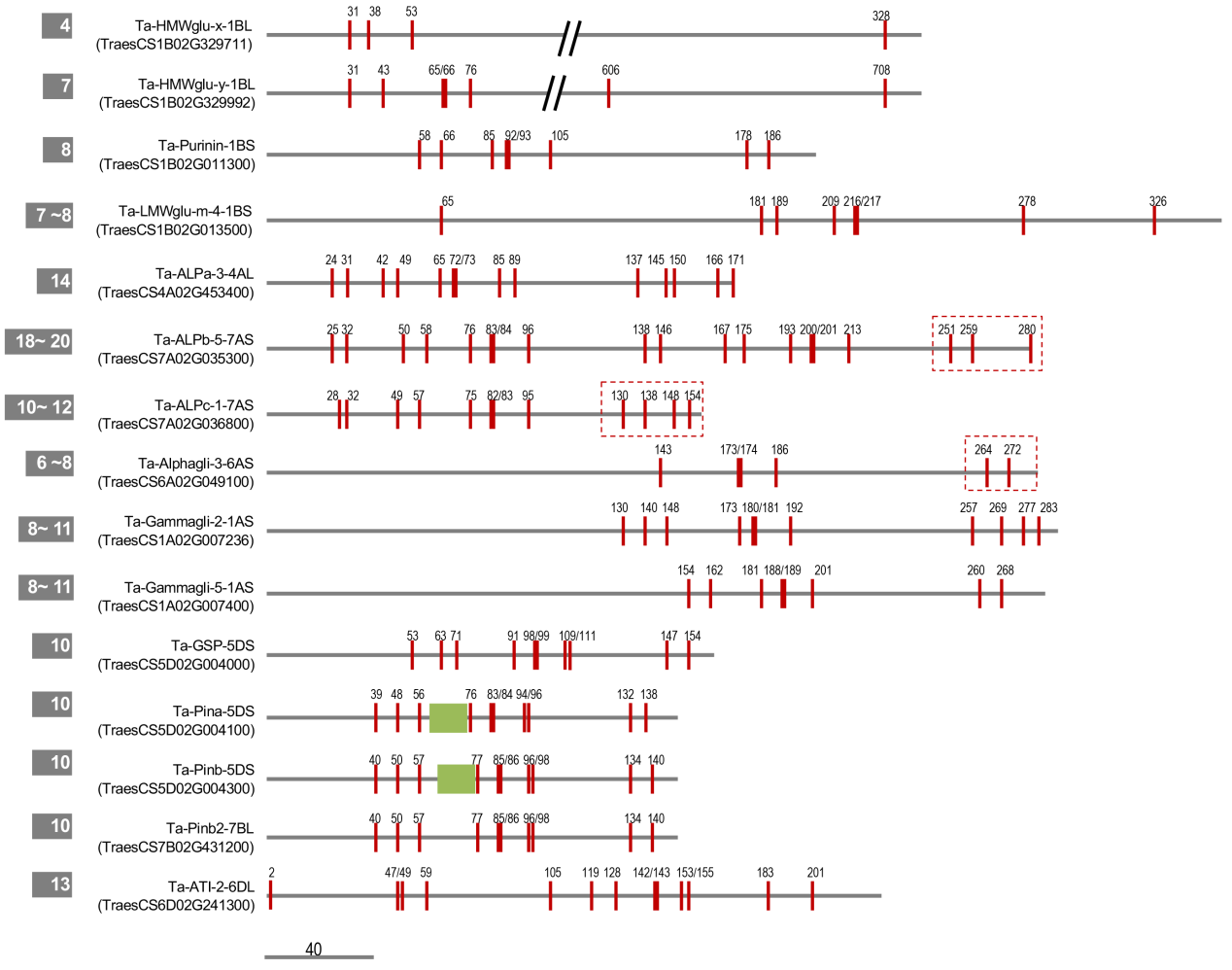
**

**Figure S4.** Comparison of cysteine residue patterns between representative proteins in the Prolamin protein families. Several families of seed storage proteins are used for the analysis, including HMW-GS (Ta-HMWglu-x-1BL, Ta-HMWglu-y-1BL), purinin (Ta-Purinin-1BS), LMW-GS (Ta-LMWglu-m4-1BS), avenin-like proteins (Ta-ALPa-3-4AL, Ta-ALPb-5-7AS, Ta-ALPc-1-7AS), α-gliadin (Ta-Alphagli-3-6AS), γ-gliadin (Ta-Gammagli-2-1AS, Ta-Gammagli-5-1AS), GSP (Ta-GSP-5DS), Purindolines (Ta-Pina-5DS and Ta-Pinb-5DS), Pinb2 (Ta-Pinb2-7BL) and ATI (Ta-ATI-2-6DL). The protein designation is consistent with Figure 2 and indicates species (‘Ta-‘), protein family (‘ALPa’), gene number (‘5-’) and chromosomal location (‘-4AL’). Cysteine residues are shown in red bar with the TRD of PINA and PINB shown in green bar. The number of cysteine residues are indicated on the left panel. Scale bar =40 amino acids.

**
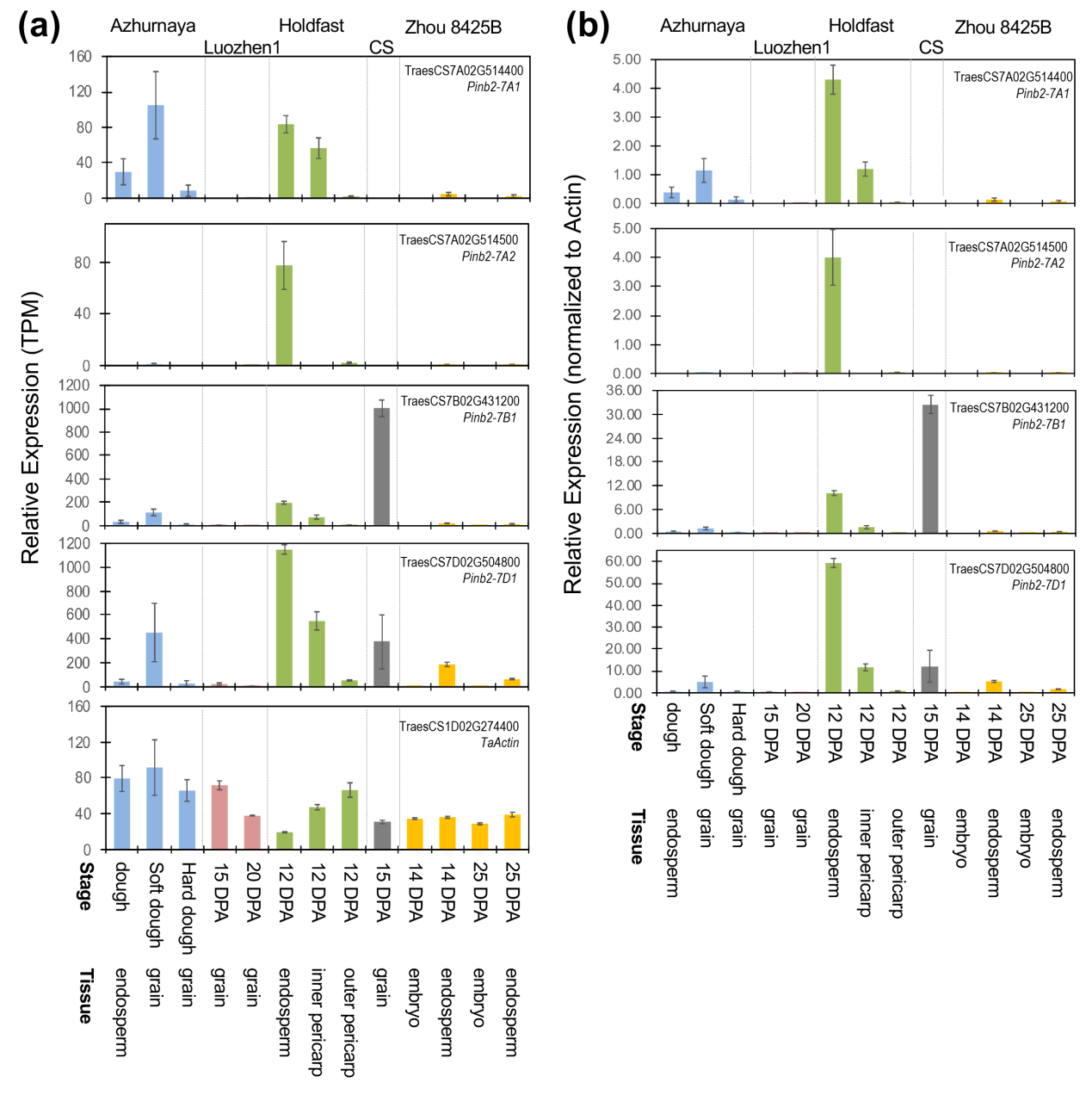
**

**Figure S5.** Expression patterns of *Pinb2* in developing wheat seeds. To visualize the expression levels (TPM) of Pinb2 across wheat varieties and stages during seed development, *Pinb2* expression in the seed tissues (including grains, embryo and endosperms) from the five RNA-seq data were plotted together with the reference gene *TaActin* **(a)** or normalized to the *TaActin*’s expression **(b)**. Wheat varieties are colored as blue (Azhurnaya), red (Luozhen 1), green (Holdfast), grey (CS) and yellow (Zhou 8425B).

**
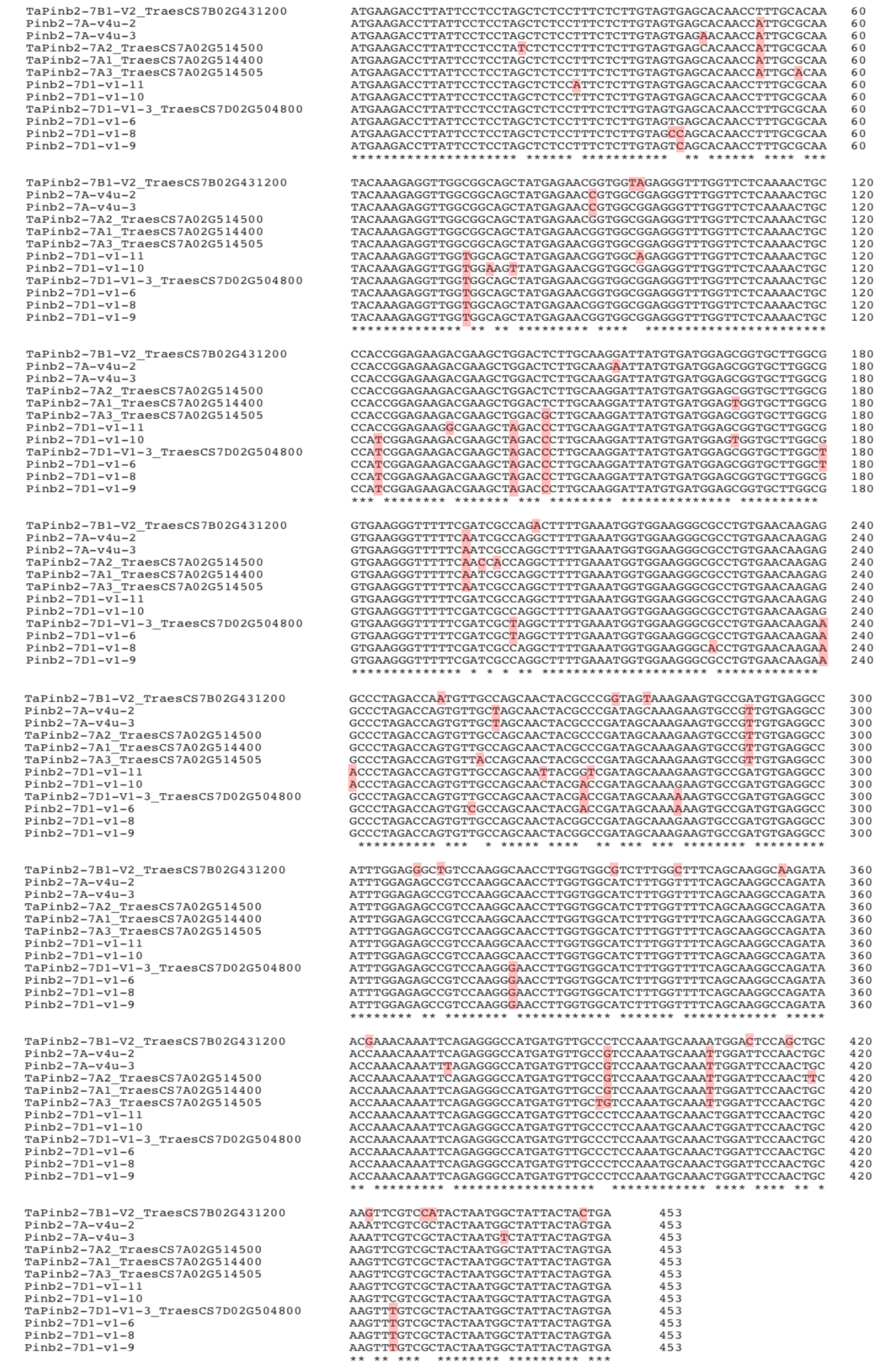
**

**Figure S6.** Sequence alignments of the new identified *Pinb2* alleles. Non-conserved nucleotides are shaded in red.

**Table S1.** The new designation of *Pinb2* genes. To be consistent with the IWGSC RefSeq v1.0 annotation, “Pinb-2v” used in previous literature is re-designated as “Pinb2” and suffixes “-7A”, “-7B” and “-7D” are used to show the chromosomal locations of the loci. The corresponding variants in cultivar Chinese Spring (CS) are shown in the Table.

| **Locus name** | **Gene name** | **Gene model** | **Previous nomenclature** |
| --- | --- | --- | --- |
| *TaPinb2-7A* | *TaPinb2-7A1* | TraesCS7A02G514400 | *Pinb-2v4.1* |
|  | *TaPinb2-7A2* | TraesCS7A02G514500 | not reported previously |
|  | *TaPinb2-7A3* | TraesCS7A02G514505 | Not reported previously |
| *TaPinb2-7B* | *TaPinb2-7B1* | TraesCS7B02G431200 | *Pinb-2v2-1* |
| *TaPinb2-7D* | *TaPinb2-7D1* | TraesCS7D02G504800 | *Pinb-2v1-4* |

**Table S2**. The gene models correspond to *Pinb2* genes.

| **Species (genome)** | **Gene Name** | **Ortholog**  **Genes in wheat** | **Gene model** |
| --- | --- | --- | --- |
| *T.urartu* (AA) | *TuPinb2-7A* | *TaPinb2-7A* | TuG1812G0700005557.01.T01 |
| *Ae.tauschii* (DD) | *AtPinb2-7D* | *TaPinb2-7D* | AET0Gv20021600.1 |
| *T.turgidum dicoccooides*  (AABB) | *TtPinb2-7B* | *TaPinb2-7B* | TRIDC7BG068420.1 |
|  | *TtPinb2-7A* | *TaPinb2-7A* | three homologous sequences putatively encoding Pinb2 copies without annotated gene models * |

Note: * The three homologous gene copies putatively encode the Pinb2 gene are located between TRIDC7AG072280.2 and TRIDC7AG072300.6.

**Table S3.** Abbreviations of the genes shown in Figure2 and S3 that presents the syntenic alignment results. [provided as Excel file]

**Table S4.** Annotation of the genes from prolamin superfamily, globulin and albumin families used in this study. [provided as Excel file]

**Table S5.** Information about the five publicly available RNA-seq datasets used for *Pinb2* expression analysis.

| **Dataset** | **Title of the dataset in Triticeae Multi-omics Center** | **Reference *** | **Project Brief Information** | **Cultivar** | **NCBI accession** |
| --- | --- | --- | --- | --- | --- |
| 1 | BCS cv-1 Development | IWGSC 2018 Science. | A development gene expression atlas was established using commercial wheat cultivar Azhurnaya for 209 RNAseq samples representing 22 tissue types from grain, root, leaf, and spike samples across multiple time points using the IWGSC RefSeq v1.0 genome. | Azhurnaya | PRJEB25639 |
| 2 | RNA-seq of pericarp of purple-grain wheat | NA | The grains of purple-grain wheat cultivar Luozhen1 at 15 and 20 DAP with or without shading treatments were used for RNA-seq using an Illumina HiSeqTM 2000 platform. | Luozhen1 | PRJEB22854 |
| 3 | Tissue layers from developing wheat grain at 12 days post-anthesis | NA | Endosperm, inner pericarp and outer pericarp manually isolated from the immature grain of wheat cultivar Holdfast at 12 DAP were used for single-end RNA-seq using illumina Genome Analyzer IIx. | Holdfast | PRJEB7795 |
| 4 | Unveiling multi-dimensional regulation of heat stress –responsive transcriptomes in wheat | NA | Plants of wheat cultivar CS were treated with heat stress. Grain and leaf samples were collected at 0m, 5m, 10m, 30m, 1h and 4h of stress treatment and used for RNA-seq analysis by illumina (HiSeq X Ten) and PacBio (RS II) Iso-seq. | CS ^#^ | PRJNA427246 |
| 5 | Expression of embryo and endosperm in developing grain | Wei et al. 2019 | Embryo and endosperm samples were manually dissected and used for pair-end RNA-seq analysis using illumina HiSeq 4000. | Zhou 8425B | PRJNA485741 |

* References are listed in the Supplementary References. # CS = Chinese Spring; NA = not applicable; DAP = Days after pollination

**Table S6.** Primers for qRT-PCR of *Pinb2*.

| Primer  Name | Sequence | Product  length (bp) | Target gene |
| --- | --- | --- | --- |
| Actin | TCTATTTTGGCCTCTCTTAGCAC | 200 bp | *Actin* |
|  | TTTCCTGTACCCCTTATTCCTC |  |  |
| Q2 | AATTCAGAGGGCCATGATGTT | 150 bp | *Pinb2* |
|  | CCCTTCCACCATTTCAACAG |  |  |
| Q | CACAATAAAGGATTTTCCAGTCAC | 187 bp | *Pinb* |
|  | GAATACCTCACCTCGCCACAT |  |  |

**Table S7**. Primers used for *Pinb2* genotyping.

| Primer name | Sequence | Product  length (bp) | Target allele/  variant |
| --- | --- | --- | --- |
| D | ATATAGAGTCCGTGATTGTATC  TCCTCTCCTCAAACATTCT | 1586 bp | *Pinb2-7D-2v1* |
| B1 | ATGCCAAGGAGCCACCACTAT  CGGGCGGCATAGAGTCAAC | 1326 bp | *Pinb2-7B-2v3* |
| B2 | GGTGTGGTCGTGTGGGGTA  ATTAGAATGTGTGTATTTATTTTGGTAT | 1793 bp | *Pinb2-7B-2v2* |
| B3 | GACTCTTATCTCTTCAGTTCATCGTC  TATTTAGAATCATCTGGTCGCACA | 1132 bp | *Pinb2-7B-2v2* |
| U | ACGGGACTCTTATCTCTTCTG  GGGAGCATAGTCTTGTCAAA | 734 bp | *Pinb2* |
| C | TGGCGTCTTTGGCTTTCAG  CACGAGTGATTTCAGTAGTAATAGCC | 135 bp | *Pinb2-7B-2v2* |

**Table S8.** *Pina*/*Pinb* and *Pinb2* genotypes for the 70 Chinese wheat varieties and their kernel hardness phenotypes.

| Variety name | *Pinb2-7B1* | *Pinb2-7D1* | *Pina* allele | *Pinb* allele | Average HI in 2016/17 | Average HI in 2017/18 |
| --- | --- | --- | --- | --- | --- | --- |
| Liaochun 9 | *2v2-1* | *2v1-4* | a | a | 25.8 | 24.9 |
| Miannong 2 | *2v2-1* | *2v1-4* | a | a | 11.8 | 16.1 |
| Miannong 4 | *2v2-1* | *2v1-4* | a | a | 19.5 | 13.7 |
| Hongqimai | *2v2-1* | *2v1-4* | a | a | 25.3 | 25.1 |
| Baidatou | *2v2-1* | *2v1-4* | a | a | 29.7 | 24.4 |
| Baiyangmai | *2v2-1* | *2v1-4* | a | a | 41.4 | 42 |
| Bonong 7023 | *2v3a* | *2v1-4* | a | a | 14.1 | 14.7 |
| Emai 18 | *2v3a* | *2v1-4* | a | a | 27.8 | 33.5 |
| Hua 2668 | *2v3a* | *2v1-4* | a | a | 26 | 20.9 |
| E 50406 | *2v3a* | *2v1-4* | a | a | 33.1 | 28 |
| E 35264 | *2v3a* | *2v1-4* | a | a | 36.6 | 30.9 |
| Huamai 0460 | *2v3b* | *2v1-4* | a | a | 21.1 | 14.8 |
| Mianmai 45 | *2v3b* | *2v1-4* | a | a | 15.1 | 16.3 |
| Yang 06G5 | *2v3b* | *2v1-4* | a | a | 21.4 | 31.9 |
| Chuanmai 50 | *2v3b* | *2v1-4* | a | a | 32.5 | 24.1 |
| Yangfumai524 | *2v3b* | *2v1-4* | a | a | 29.7 | 19.3 |
| Aifeng 3 | *2v3b* | *2v1-3* | a | a | 25.3 | 26.1 |
| Emai1 4 | *2v3b* | *2v1-4* | a | a | 32.2 | 20 |
| Zhengmai 101 | *2v3b* | *2v1-6* | a | a | 45.5 | 50.4 |
| Baimangcao | *2v3b* | *2v1-4* | a | a | 30.1 | 29.3 |
| Hongmai | *2v3b* | *2v1-4* | a | a | 23.7 | 14.7 |
| Hongxiaomai | *2v3b* | *2v1-3* | a | a | 33.8 | 35.2 |
| Huamai 0480 | *2v2-1* | *2v1-4* | a | b | 44.7 | 51.8 |
| Wanmai 50 | *2v2-1* | *2v1-4* | a | b | 38.1 | 38.8 |
| Xinmai 13 | *2v2-1* | *2v1-4* | a | b | 39.2 | 45.3 |
| Jimai 36 | *2v2-1* | *2v1-4* | a | b | 66.5 | 70.5 |
| Xinchun 7 | *2v2-1* | *2v1-4* | a | b | 65.8 | 66.2 |
| Zhengmai2441 | *2v2-1* | *2v1-4* | a | b | 63.9 | 61 |
| Baiyumai | *2v2-1* | *2v1-3* | a | b | 70.7 | 65.5 |
| Xiangmai 35 | *2v3a* | *2v1-4* | a | b | 69.3 | 68.3 |
| Emai 895 | *2v3a* | *2v1-4* | a | b | 68.6 | 76.9 |
| Emai 793 | *2v3a* | *2v1-4* | a | b | 66 | 78.1 |
| Emai 048 | *2v3a* | *2v1-4* | a | b | 75 | 76 |
| Emai 195 | *2v3a* | *2v1-4* | a | b | 69.1 | 81.4 |
| Emai 170 | *2v3a* | *2v1-4* | a | b | 48.9 | 47.6 |
| Jinmai 47 | *2v3b* | *2v1-3* | a | b | 52.8 | 63.7 |
| Mianmai 41 | *2v3b* | *2v1-4* | a | b | 72.4 | 68 |
| Zhen 05185 | *2v3b* | *2v1-4* | a | b | 69.3 | 71.4 |
| Su 8637 | *2v3b* | *2v1-4* | a | b | 50.2 | 48.6 |
| Wanke 06229 | *2v3b* | *2v1-6* | a | b | 57.1 | 60.7 |
| Yang 87-158 | *2v3b* | *2v1-4* | a | b | 60 | 57 |
| Ning 6E125 | *2v3b* | *2v1-4* | a | b | 65.6 | 70.6 |
| Jimai 26 | *2v3b* | *2v1-3* | a | b | 61.2 | 56.7 |
| Jimai 107 | *2v3b* | *2v1-6* | a | b | 48.2 | 58.3 |
| Ganchun 4589 | *2v3b* | *2v1-4* | a | b | 63.2 | 66.9 |
| Hengmai 1 | *2v3b* | *2v1-3* | a | b | 59.6 | 67.4 |
| Jimai5099 | *2v3b* | *2v1-4* | a | b | 61.9 | 63.2 |
| Jin 311 | *2v3b* | *2v1-4* | a | b | 62 | 64.7 |
| Jingmai 103 | *2v3b* | *2v1-4* | a | b | 82.9 | 74.3 |
| Zhengmai 004 | *2v3b* | *2v1-3* | a | b | 62.8 | 62.1 |
| Zhengmai 318 | *2v3b* | *2v1-3* | a | b | 58.6 | 55.4 |
| Baimai | *2v3b* | *2v1-3* | a | b | 60.5 | 62.3 |
| Xiaoyan 22 | *2v3c* | *2v1-4* | a | b | 77 | 71 |
| Changwu 131 | *2v3c* | *2v1-4* | a | b | 52.5 | 49.2 |
| Zhengmai1308 | *2v3c* | *2v1-4* | a | b | 66 | 72.9 |
| Zhengmai 122 | *2v3c* | *2v1-3* | a | b | 65.7 | 72.1 |
| Fengkang 10 | *2v2-1* | *2v1-4* | a | p | 45 | 50.8 |
| Baishuimai | *2v2-1* | *2v1-3* | a | p | 75.4 | 72.8 |
| Baiqimai | *2v2-1* | *2v1-4* | a | p | 81.7 | 73.1 |
| Honglimai | *2v2-1* | *2v1-4* | a | p | 69.3 | 82.1 |
| Honghuawu | *2v2-1* | *2v1-3* | a | p | 81.3 | 88.2 |
| Baicanmai | *2v2-1* | *2v1-3* | a | p | 81.1 | 75 |
| Yang 9856 | *2v3b* | *2v1-4* | a | p | 73.6 | 72.5 |
| Xinmai 8 | *2v3b* | *2v1-4* | a | p | 65.8 | 63.4 |
| Yang 12G16 | *2v3b* | *2v1-4* | a | p | 80.8 | 74.5 |
| Laoqimai | *2v3b* | *2v1-6* | a | p | 65.3 | 66.6 |
| Hongmangmai | *2v3b* | *2v1-3* | a | p | 78.4 | 71.9 |
| Hongmangmai | *2v3b* | *2v1-4* | a | p | 83.8 | na |
| Zhongmai1142 | *2v3b* | *2v1-4* | a | p | 71.1 | 65 |
| Shan 357 | *2v3c* | *2v1-4* | a | p | 68 | 83.2 |

“na” = not applicable.

**Supplementary References**

Chen, F.; Beecher, B.S.; Morris, C.F. Physical mapping and a new variant of *Puroindoline b-2* genes in wheat. *Theor. Appl. Genet.* 2010, 120, 745-751.

Chen, F.; Xu, H.X.; Zhang, F.Y.; Xia, X.C.; He, Z.H.; Wang, D.W.; Dong, Z.D.; Zhan, K.H. et al. Physical mapping of *puroindoline b-2* genes and molecular characterization of a novel variant in durum wheat (*Triticum turgidum* L.). *Mol. Breed*. 2011, 28, 153–161.

International Wheat Genome Sequencing Consortium (IWGSC). Shifting the limits in wheat research and breeding using a fully annotated reference genome. *Science*. 2018, 361, eaar7191.

Wei, J.; Cao, H.; Liu, J.D.; Zuo, J.H.; Fang, Y.; Lin, C.T.; Sun, R.Z.; Li, W.L.; Liu, Y.X. Insights into transcriptional characteristics and homoeolog expression bias of embryo and de-embryonated kernels in developing grain through RNA-Seq and Iso-Seq. *Funct. Integr. Genomics*. 2019, 19, 919-932.
